# Large-scale parallel alignment of platelet-shaped particles through gravitational sedimentation

**DOI:** 10.1038/srep09984

**Published:** 2015-05-18

**Authors:** Sebastian Behr, Ulla Vainio, Martin Müller, Andreas Schreyer, Gerold A. Schneider

**Affiliations:** 1 Institute of Advanced Ceramics, Hamburg University of Technology, Hamburg, Germany; 2 Institute of Materials Research, Helmholtz-Zentrum Geesthacht, Geesthacht, Germany

## Abstract

Parallel and concentric alignment of microscopic building blocks into several orders of magnitude larger structures is commonly observed in nature. However, if similarly aligned structures are artificially produced their thickness is generally limited to just about one or two orders of magnitude more than the dimensions of the smallest element. We show that sedimentation provides a promising approach to manufacture solid materials consisting of well-aligned platelet-shaped particles while being more than 30 000 times thicker than the individual particle. Such sediments contain up to 28 vol% of particles without any further treatment and can be densified to 67 vol% particle fraction by subsequent unidirectional pressing. The degree of orientation of the platelet-shaped particles within the sediments was tracked by high-energy X-ray diffraction measurements. The Hermans orientation parameter, a statistical measure of the quality of alignment, was determined to be 0.63 ± 0.03 already for as-sedimented samples while the standard deviation of the orientation distribution of particles, another measure of average misalignment, was found to be (21.5 ± 1.4)°. After pressing, these values further improved to (0.81 ± 0.01) and (14.6 ± 0.4)°, respectively. Such quality of alignment competes with, if not even exceeds, values reported in the literature.

Parallel-aligned platelet-shaped particles, concentrically-aligned tubes, and structures based upon such ordering can be found in nature as well as in man-made engineering. From a technical point of view, the functions of such materials vary from purely mechanical, optical, and electrical all the way to their combinations into multifunctional materials. Parallel alignment in engineering approaches can usually be found on the macro-scale with some of the best-known examples being brick walls, parallel-plate capacitors, and structural laminates. Nature, on the contrary, normally utilizes ordering on sub-micrometre length-scales and in a hierarchical manner with some examples being – amongst others^1^,^2^,^3^ – enamel in teeth^4^,^5^, spicules of some marine sponges^6^,^7^,^8^, bone^9^,^10^,^11^ and nacre^12^,^13^,^14^,^15^.

In past years, nacre, also known as mother-of-pearl, has taken a special position in the field of well-aligned material structures as it bridges the gap between nature and engineering by being one of the most-investigated examples for bio-inspiration and biomimetics^16^. The reason for this is the rather simple brick-and-mortar-like structure and the fact that nacre develops almost planar with just a slight curvature when compared to, for example, spicules and bone. Thus, it can be mimicked using flat surfaces as templates. Furthermore, nacre exhibits a unique combination of high strength and high toughness^12^, properties, which are generally hard to achieve simultaneously^17^. The reason for this unique combination is believed to be mainly due to the as-mentioned brick-and-mortar microstructure of nacre^12^,^15^.

As a consequence, several scientific groups have worked on manufacturing of synthetic materials inspired by nacre and its microstructure^16^,^18^,^19^,^20^,^21^,^22^,^23^,^24^,^25^,^26^,^27^,^28^,^29^,^30^,^31^,^32^,^33^,^34^,^35^. However, it is evident that despite the huge number of publications in the field of nacre-inspired composites, there are almost no reports on bulk-sized samples consisting of highly concentrated micro-scaled, platelet-shaped particles in a well-aligned, parallel ordering over centimetres in thickness. Almqvist *et al.*^36^ analysed the aligning ability of several different processing techniques on platelet-shaped particles. They also quantitatively measured the quality of alignment and rated the different techniques according to their quality of alignment. However, Almqvist *et al.* did not provide any information on particle concentration or achievable sample thickness. Oner Ekiz *et al.*^21^ used pressure-assisted slip casting to prepare nacre-like bulk composites. They achieved a remarkable improvement in mechanical properties compared to their matrix material and reached a particle fraction of up to 60 vol% with a modest quality of alignment. However, the paper does not quantitatively measure the alignment and its evaluation is limited to electron microscopy images. Methods based on freeze-casting have shown great potential for superior mechanical performance^22^,^23^,^28^,^32^,^33^. While they are promising for yielding larger samples, though, they mainly rely on domains of identical alignment of the lamellae that are typical for the process. However, those domains are generally not bigger than a few millimetres in every spatial extension^37^, a fact that results in similarly small sample dimensions. This problem has only recently been solved by Bouville *et al.*^38^ and to the best of our knowledge their findings have not yet been applied to composites manufacturing. Furthermore, most of the publications on freeze-cast nacre-like composites similarly lack a quantitative assessment of structural alignment.

Other methods that have been identified to give best results with respect to alignment of micro- and nano-sized particles (*e.g.* shearing or layer-by-layer-like assembly^19^,^20^,^24^,^25^,^26^,^36^,^39^,^40^,^41^,^42^) can be used most efficiently for thin films and low filler concentration. Those films may in turn be suitable for composite lay-up^26^,^42^ but are difficult to transfer into centimetre-sized bulk samples. On the other hand, laminating sub-millimetre-sized ceramic plates has been reported to yield excellent mechanical performance, but fracture mechanics were limited to large scale phenomena rather than sub-micron observations made with natural nacre^19^,^20^,^34^,^35^. Again, most of these works do not quantify the quality of alignment.

With this work, we show that gravitational sedimentation, despite its rating as a rather weak solution for parallel-alignment until now^36^, can easily yield samples with good alignment of micrometre-sized, platelet-shaped particles on centimetre thickness scales. In addition, the planar extensions of sedimentation vessels are theoretically unlimited, giving excellent conditions for application in large-volume industrial processes. Furthermore, gravitational sedimentation by itself can be rated as highly energy-effective and thus promising for green manufacturing. In addition to our results, these claims can, amongst others, be substantiated by references to the natural formation of sedimentary rock, resulting in thick and broad occurrence of aligned textures formed through a similar mechanism^43^.

In order to quantify the quality of alignment of the particles in our sediments in a statistically meaningful manner, we used high-energy X-ray diffraction (HEXRD). Being a non-destructive method, it allows us to characterize samples as they have been prepared without further processing that could influence the results. HEXRD gives an accurate, spatially resolved characterization of the shape of the orientation distribution, of the average misalignment, and of the orientation degree of the particles.

## Results

Micrometre-sized, single-crystal alumina platelets were sedimented in solutions of various concentrations of polyvinyl butyral (PVB) in ethanol (EtOH). Resulting sediments were then either characterized directly following sedimentation or were further densified by unidirectional pressing at elevated temperature.

**Figure 1:**
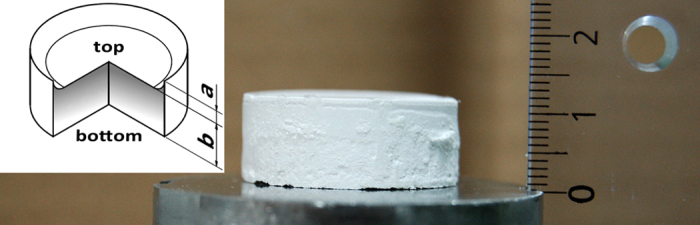
Non-pressed sediment sample right after demolding. The scale to the right is a centimeter-scale with each dash representing a length of 1 mm. The sample shown here has a height of 11 mm but the sample height could be scaled by changing the particle concentration in suspension or the filled height of the sedimentation vessel. The schematic on the left indicates typical dimensional and structural characteristics of our sediments: All sediments exhibit a meniscus of height *a* on a cylindrical body with a thickness *b*. For X-ray analysis, only top sections of thicknesses up to 2 mm were used. Furthermore, the schematic illustrates the distribution of polymer inside the sediments with higher fractions towards the top (darker grey) and lower fractions toward the bottom (light grey).

**Figure 2:**
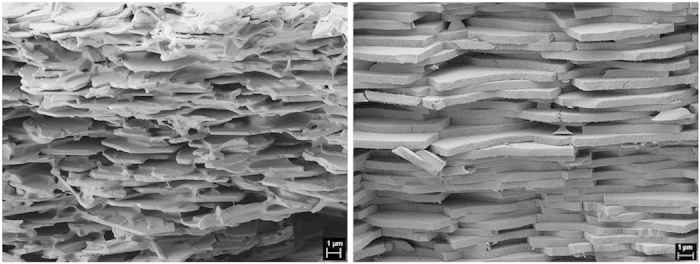
Comparison of artificial nacre as prepared by sedimentation according to the process described in this work (left) and a typical detail of a fracture surface in natural nacre (right). The section from the artificial sample shown here is located at the very top of the sediment and consists of about 28 vol% particles, 5 vol% PVB and 67% porosity.

In general, complete sedimentation was obtained after one to three days, depending on the viscosity of the suspension. Subsequent drying required another one to three weeks for the experimental setup used in this work. After demolding, all sediments have a thickness of about 10 to 12 mm (see fig. 1). Fig. 2 illustrates the degree of alignment in the upper part of a representative sediment and the structural similarity to natural nacre. The viscosity of the different suspensions varied broadly with polymer concentration, ranging between approximately 1.5 mPa·s and 120 mPa·s. However, irrespective of the viscosity and polymer concentration in solution, the sedimentation process resulted in arrays of platelets that are well aligned horizontally (*i.e.* parallel to the sediment surface) with only small, localized distortions. Comparing all samples, no significant structural differences were observed between them. Visual analysis by scanning electron microscopy (SEM) at several positions over the thickness of the sediments shows low misalignments at the lower- and uppermost regions of the sediments but slightly higher distortion in between.

**Figure 3:**
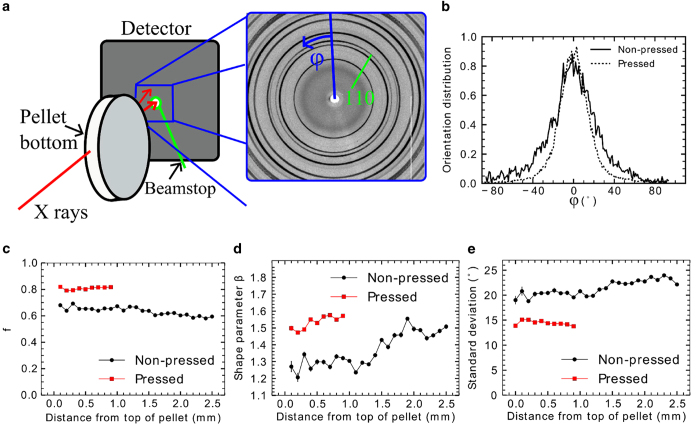
Graphic representation of the setup for X-ray diffraction and the corresponding results. **a**) Schematic presentation of the HEXRD measurement geometry (not in scale) and an example of a portion of a collected XRD pattern. For convenience of calculations of parameters, the zero of azimuthal angle *φ* is defined here to be at a maximum of the 110 reflection. **b**) Orientation distributions of particles at 0.5 mm and 2.1 mm depth from the pellet’s top for a pressed and a non-pressed pellet, respectively, as a function of azimuthal angle. The variation seen in the distribution is mostly due to sampling of platelets rather than statistical variation of the intensity. **c**) Hermans orientation parameter, **d**) orientation distribution shape, and **e**) standard deviation of the orientation distribution as a function of distance from the top of the sample for the particles of a non-pressed and pressed pellet. The error bars represent 1σ uncertainties obtained from the statistical uncertainties of the intensities using Monte Carlo error analysis.

**Figure 4:**
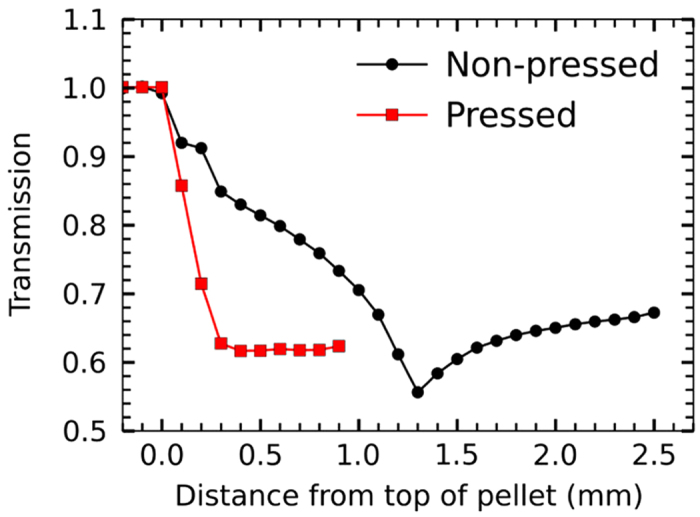
X-ray transmission profiles of a non-pressed and a pressed pellet. Measurements were done from the side of the pellet at X-ray energy of 98.7 keV.

Figure 3a and b show the geometry for the high-energy X-ray diffraction (HEXRD) measurements and the typical orientation distributions obtained for the samples under investigation. Those experiments were applicable since all particles were single-crystalline with identical crystallographic orientation, *i. e.* their plane normal corresponding to the c-direction [0 0 1] in alumina. The distributions are an average over the 30 mm diameter of the sediment pellets within a 0.1 mm slice from the side of the pellet. XRD data were only collected for the polymer-stabilized top sections of the sediments (see comments on mechanical properties and polymer distribution below). Based on the XRD data, the Hermans orientation parameter (fig. 3c, Eq. 4), the average shape of the orientation distribution (fig. 3d, Eq. 6), and the standard deviation of the orientation distribution (fig. 3e, Eq. 7) of the particles within this slice from the top downwards were determined. By tracking these, the following can be observed. In case of the non-pressed sample under investigation, the orientation degree slightly increases towards the top of the pellet. At the same time, the shape of the orientation distribution becomes sharper and approaches the Laplace distribution. However, as can be seen from the transmission graph of the pellet in fig. 4, the top surface of the pellet is not well-defined and most of the signal up to 1.3  mm distance from the top of the pellet could come from a rim formed by the liquid meniscus during the drying process. The standard deviation of the measured distribution, representing the average misalignment of particles, is found to be (21.5 ± 1.4)°, and the corresponding Hermans orientation parameter rates at 0.63 ± 0.03 for the non-pressed sample. In case of the pressed pellet, obtained by pressing the solid top section of a sediment, the orientation degree is significantly higher than for the non-pressed pellet with a standard deviation of the distribution of only (14.6 ± 0.4)° and a Hermans orientation parameter of 0.81 ± 0.01. The shape of the orientation distribution changes less across the pressed pellet.

**Figure 5:**
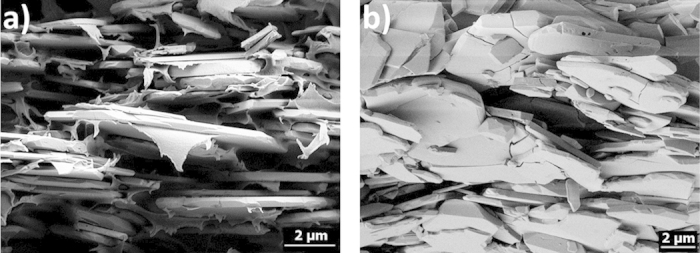
SEM images of fracture surfaces of non-pressed and pressed sediments. **a**) Typical thicknesses of interparticle layers of PVB and evidence of ductile tearing events during fracture close to the top of non-pressed sediments. **b**) SEM image of a fracture surface at the bottom of a pressed sediment. It can be clearly seen that several particles are partially cracked or fully fractured themselves, a condition that can most likely be attributed to the pressing procedure since fracture surfaces of non-pressed sediments do not show evidence of particle cracking.

Regarding the interaction of polymer and particles, fig. 5a shows a part in the upper third of a non-pressed sediment prepared from a suspension with 0.5 wt% PVB in solution (corresponding to 10 wt% related to particles only). Besides slight misalignments of the platelets with respect to each other, one can clearly see PVB around and between the platelets. This PVB seems to attach well to the particles, resulting in nacre-like stacks of platelets with thin adhesive films in between. The latter are thereby torn in a rather ductile manner. Nevertheless, despite the good alignment of particles and clear evidence of PVB, significant pore volume is clearly visible.

By applying thermogravimetric analysis (TGA), pycnometry, and equation (1) to (3), 2, 3 from the methods section to evaluate polymer content and porosity, the actual fractions of particles, polymer, and porosity can be obtained. Within the limits of statistical variation, the particle concentration in all non-pressed samples is constant, irrespective of the polymer concentration in solution. In all non-pressed samples, the particles occupy an average of (25 ± 3) vol%. The remaining volume is mainly occupied by porosity (always more than 60%) with an increasing fraction of polymer for increasing concentrations in the corresponding suspension. Regarding the distribution of polymer and porosity throughout the sediments, a strong gradient from top to bottom is observable. While the top of all samples is enriched in polymer, the bottom is highly depleted from it (see fig. 1). Meanwhile, the particle concentration is almost constant throughout each individual sediment. Its difference decreases by an average of only 0.3 vol% from bottom to top. A visual impression of the differences in polymer concentration can be gained by comparing fig. 5a and fig. 5b with fig. 5a showing a section closer to the top of a sample while fig. 5b illustrates the situation close to the bottom. Although the images are hardly comparable due to different treatments, it is obvious that fig. 5b, showing a section closer to the bottom, exhibits a much lower fraction of polymer.

Upon pressing, the whole structure is densified. In the pressed samples, the amount of porosity and polymer together decreases to approximately (35.0 ± 1.9) vol% over all polymer concentrations tested as the particle concentration increases to about (65.0 ± 1.9) vol%. The gradients in concentration thereby persist. From SEM observations, it can furthermore be concluded that pressing also causes particle fracture (compare fig. 5b).

Despite the high particle concentration, the mechanical performance of all samples is rather poor with microhardness and Young’s modulus only ranging up to 5 MPa and 5 GPa, respectively. This especially holds for the lower, polymer-depleted zones closer to the bottom of the sediments that have higher porosity.

## Discussion

The results show that a high degree of alignment of multiple platelet-shaped particles can be obtained by simple gravitational sedimentation. The fact that the structure seems to be unaffected by the changes in viscosity investigated in this work suggests that no critical changes in the sedimentation process occur within this range. This conclusion in turn is in full agreement with the results published by Liu and Joseph^44^ as well as Nie *et al.*^45^ who measured and simulated movement and orientation of different elongated objects during sedimentation and correlated the respective behaviour with the Reynolds number describing this process. Their results suggest that sedimentations associated with Reynolds numbers below one lead to a constant vertical orientation of such objects during the process. With Reynolds numbers increasing above one, however, the orientation of particles during sedimentation changes from strictly vertical to strictly horizontal and finally to tumbling chaotic motion. For the conditions given by this work and settling velocities as well as Stokes’ diameters according to Bernhardt 46, for all samples at hand, the particles reach Reynolds numbers far below one. Hence, they should all sediment in the same manner, *i.e.* in a vertical edge-on orientation, and no changes are to be expected on this account. Right after contact to the bottom of the vessel or already formed sediment, the particles topple over into the mainly horizontal orientation which was observed in X-ray and SEM analysis. This reorientation is driven by gravity and overturning moments that originate in non-symmetric positioning of the just sedimented particles and by impacts from subsequently sedimenting ones. Effects counteracting this motion are contacts with already stationary particles and drag forces from the liquid. The effect of drag changes with viscosity^47^ but the above mentioned absence of significant structural alterations suggests that this effect is negligible in our case.

SEM observations showed that the quality of alignment is excellent at the very top (*i.e.* last particles to sediment) and bottom layers (*i.e.* first layers to sediment) of the sediments compared to the centre part. This may be partially explained by stacking faults, which add up over thickness. For the first layer of particles, mainly surface roughness and waviness of the surface that they sediment on determine the degree of misalignment between the individual particles. In addition, it would be possible for two or more sedimenting particles to lean against and upon each other after reaching the bottom of the vessel, forming defects similar to a house of cards. This is especially likely due to the above mentioned vertical orientation during sedimentation and edge-on landing expected for Reynolds numbers below one, combined with the proof of a preferably horizontal alignment in the sedimented samples given by SEM and XRD results. Consequently, an implied transition occurred between vertical and horizontal orientation that all particles have to undergo during settling. The second and further layers of particles are then affected by the layers and defects underneath, including the formation of new defects by settling on edges between layers of different height. As a consequence, stronger misalignment in the middle region is expected. However, this stacking of defects cannot explain the improvement of alignment close to the top of the sediment. Here, we believe that the interface between air and solvent has an aligning effect. Starting from the top, this interface advances through the sediment during drying and the surface tension reorients particles it passes tangentially to the interface. Such effects can be observed in Langmuir-Blodgett-like-processes^48^,^49^ and previous studies have even actively used them to prepare thin foils of nacre-inspired materials^25^. For increasing distance to the top of the sample, however, particles become more constrained in their mobility due to an increasing number of layers above them, and the effect diminishes.

Regarding a quantitative rating of the results, HEXRD provides an extremely helpful tool. While SEM analysis always needs well and carefully prepared surfaces – and is limited to information comprised in them – HEXRD gives an averaged picture of the whole sample volume without excessive requirements in sample preparation. Referring to the standard deviation of the orientation distribution as a measure for misalignment and the Hermans orientation parameter, the orientation degree remains fairly constant throughout the investigated sediment thickness. This confirms the findings from SEM, where rather good orientation with some noticeable defects was observed. Pressing obviously further improves the alignment and densifies the whole structure. Using pycnometry and TGA, we identified a remarkable reduction of porosity along with an increase in particle concentration. The amount of (65.0 ± 1.9) vol% of particles is a value that has hardly been achieved in nacre-like composites to date^16^. This increase in particle concentration by pressing is most likely directly related to the enhanced alignment that was observed by HEXRD (from approximately ±21.5° standard deviation for horizontal alignment in the as-sedimented sample to about ±14.6° after pressing). Particles that are slightly misaligned by leaning against others can and do break under the bending stresses generated by uniaxial compression (see fig. 5b). In this way, pores fill up with particle fragments, and all particles rearrange in the most space-efficient manner. From a mechanical point of view, however, the reduction of aspect ratio resulting from fragmentation would impair the stress transfer between particles and the matrix surrounding them^50^,^51^. Furthermore, a fragmentation in this way would generate not only a lower aspect ratio but also a broader distribution of it, assumingly causing early failure of the matrix in addition to a large scatter in strength values.

The fragmentation caused by pressing may also be one reason why the mechanical performance of the samples does not noticeably improve upon pressing. For the as-sedimented samples, the porosity definitely drastically impairs the mechanical performance and would hinder an immediate application in mechanically challenging areas. However, it could also open up opportunities for filtering processes and for acoustic and thermal isolation. Furthermore, given the rather fragile nature of the samples, it is also evident how valuable the HEXRD method is for structural characterization. Since it allows for non-destructive, full-depth penetration measurements all the way through the 30 mm diameter of the samples, it provides comprehensive and non-selective orientation data without the need of any further sample preparation. In this way, even fragile samples can be analysed without preparation artefacts.

A gradient in polymer content over thickness was identified in the SEM observations and by TGA. The reason for this can be found in the drying process. Due to the confinement inside the sedimentation tubes that were used, the drying takes place in a unidirectional manner solely through the top of the sample. In this way, top layers will dry first and precipitated PVB remains but does not completely fill all voids left by the solvent. The resulting porosity causes capillary forces to act on the polymer solution still remaining in lower layers and parts of this remaining solution are drawn into the upper layers where they dry as well. However, capillary forces can only support replenishment up to certain heights with respect to the surface of the liquid reservoir. As a consequence, the upper layers still dry but end up with an enrichment in precipitated polymer. This additional polymer in turn is no longer available for lower layers, causing depletion in these regions. Such a gradient in polymer distribution may be applicable for selective processing of composites to tailor their mechanical response, especially when a second polymer would be used to infiltrate the still existing porosity inside the samples. The resulting mechanical response of such composites would also exhibit a gradient, providing the opportunity for biomedical applications in transient regions between tendons and bones, as suggested by Genin *et al.*^52^ and Libanori *et al.*^53^. In these transient regions, the Young’s modulus of the natural material changes by orders of magnitude over short distances^52^, a behaviour not yet successfully mimicked in commercially available materials. The particle concentration was found to change just slightly over the sediment thickness. Generally, the existence of such a gradient could be anticipated due to well-known particle separation by density and effective (*i.e.* Stoke’s) diameter^54^,^55^ and one might predict a more pronounced effect. However, the sedimentation takes place out of a homogeneous suspension, in which the particles do not only differ in size but also in their remaining sedimentation length. Hence, smaller and bigger particles reach the sediment simultaneously in case of matching ratios of sedimentation length and rate and yield a polydisperse particle size distribution for the first sediment layers. A comparative example for similar phenomena can be found in clastic sedimentary rock formations which, under the influence of a constant polydisperse feed of deposit, also show a polydisperse size distribution of clast material^43^. Yet, in a closed system like the suspensions at hand this can only hold as long as the system is still homogeneous, a state that is disturbed by the particle separation mentioned at the beginning. Consequently, the particle size distribution shifts over the sediment thickness and gradually changes from a polydisperse to a monodisperse character from bottom to top. With packing densities generally decreasing for narrowing particle size distributions^56^, this leads to a decrease of particle fraction from the bottom to the top of sediments. However, the homogeneous initial state present for the experiments at hand in turn weakens this effect and results in just slight differences.

In conclusion, we showed that the use of sedimentation in low-viscosity liquids may overcome the problem that brick-and-mortar-like structures could only be prepared in a well-aligned manner when being limited to thin foils. Sedimentation is able to overcome this limitation as it yields highly ordered arrangements of platelet-shaped particles with volume fractions of up to 28% over thicknesses well above 10 mm. Subsequent pressing further enhances this concentration to 67 vol% and thus opens up a completely new opportunity in composite manufacturing. High-energy synchrotron XRD allows the analysis of ordering in such structures qualitatively and quantitatively in an unselective manner within seconds.

Reviewing the sedimentation and drying processes for the sediments described here, apparently rather long processing times may constrain industrial application. With the constrains given in this work, namely unidirectional drying and a relatively narrow sedimentation tube, processing time will definitely be a limiting factor. Processing times could presumably be shortened using wider vessels given the theoretically unlimited planar extension of the process. Such a change in conditions would not only increase the surface area, and hence evaporation through it, but it would also diminish the slowing and coagulating effect vessel walls may have on particles moving along them^57^. In addition to a widening of the sedimentation vessel, additional measures could be taken to enhance sediment drying, *e.g.* by controlled ventilation or application of vapour-permeable membranes to achieve multi-directional drying conditions.

## Methods

**Figure 6:**
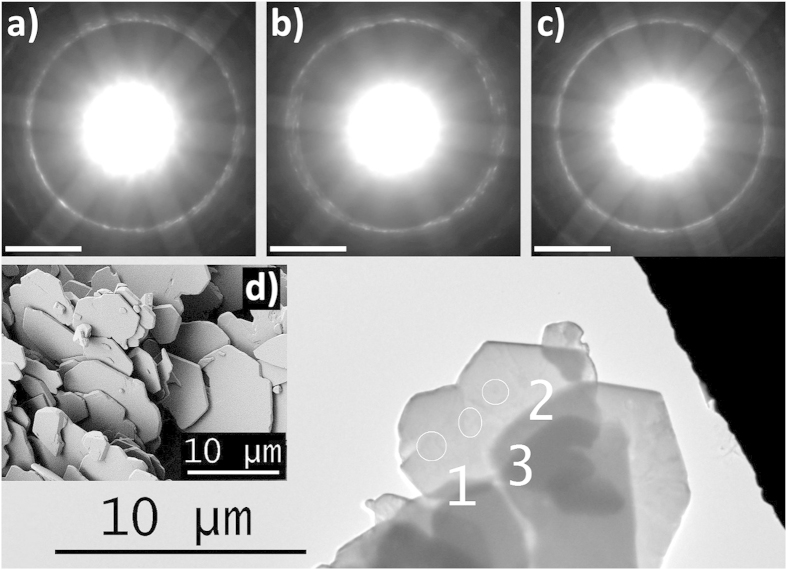
SEM and TEM images of the alumina particles used in this study. The background shows a real space image of a set of particles analysed by TEM. The Kikuchi patterns in **a**), **b**), and **c**) belong to the spots labelled 1, 2, and 3, respectively. The congruency of the Kikuchi patterns indicates single-crystallinity. **d**) A real space image of the particles from SEM. The equivalent spherical diameter of the particles as obtained by Fraunhofer diffraction is approximately 9.9 μm (d_50_).

Prior to sedimentation, ethanol-based suspensions containing one volume percent of alumina platelets and varying amounts of dissolved polyvinyl butyral (PVB) were prepared as follows. In the first step, PVB powder (Mowital B60HH, Kuraray Europe GmbH, Germany) was hand-stirred into denatured (99%) ethanol and dissolved at room temperature during 24 hours of rolling on a tumbling roller mixer. Concentrations of 0 to 9.95 wt% of PVB with corresponding dynamic viscosities of 1.5 to 121.1 mPa∙s (rotational rheometer Kinexus pro, Malvern Instruments GmbH, Germany; double-gap geometry, all slightly viscoelastic behaviour) were used in this step to alter the viscosity of solution and suspension and to change the final strength of the fully dried sediment. Afterwards, 1 vol% of platelet-shaped aluminium oxide powder (about 10 μm in diameter, 300 nm thick, RoneFlair^®^ White Sapphire, Merck KGaA, Germany), shown in fig. 6d, was added and shear-blended with the PVB solution, using a conventional blade agitator, followed by another 24 hours on the tumbling roller mixer to assure good homogenization and degassing while avoiding evaporation of ethanol. The readily-homogenized suspension was then poured into custom-made PTFE sedimentation tubes with flat bottom (average surface roughness *R*_*a*_ ≈ 1.5 μm) and left for slow sedimentation inside a fume hood. The inside of the tubes thereby had a suspension-filled height of 20 cm and a diameter of 30 mm. After full sedimentation of the particles as indicated by unobscured clarity of the excess liquid, the latter was pipetted off until only the wet sediment remained. This was then left undisturbed for drying at room temperature inside a fume hood for at least one week. The actual drying time for every sediment depended on the amount of PVB dissolved in the suspension since its precipitation during drying, starting from the top of the sediment and propagating through the sediment, progressively hindered further ethanol evaporation. Finally, the about 12 mm thick sediments were carefully removed from the sedimentation tubes and then cut and polished for characterization. In addition to direct preparation for characterization, some samples were densified by unidirectional pressing. For pressing, samples were first preloaded with 14.5 MPa at 120 °C for one hour, then pressed with 135 MPa at 120 °C for another 30 minutes and finally slowly cooled to room temperature by convection with the environment at constant deformation.

Ceramic content and porosity have been evaluated by combined use of thermogravimetric analysis (TGA) and helium-pycnometry. Due to low strength, measurements by Archimedes’ principle proved to be not reproducible for the samples at hand. With the data measured by pycnometry and TGA, the following set of equations was used to determine solid volume fractions and porosity. By rule of mixture for a porous two phase composite, its density is


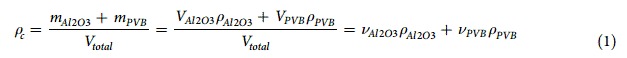


with *ρ*, *m*, *V* and *ν* being mass density, mass, volume and volume fraction and indices Al_2_O_3_, PVB and *c* standing for the alumina particles, PVB, and the whole composite, respectively. In the final product, the total porosity *p* and all solid volume fractions must sum up to unity, giving


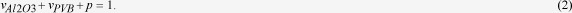


Furthermore, one can utilize that


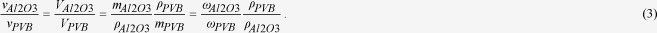


Here, *ω* indicates the mass fraction. With (1) to (3), volume fractions and porosity were easily determined from corresponding measurements. The densities of the pure alumina particles and PVB were individually measured by helium-pycnometry and amount to (3.930 ± 0.017) g/cm^3^ and (1.115 ± 0.002)g/cm^3^, respectively (n = 5, 70 measurements each, arithmetic average ± standard deviation). Mass fractions of alumina platelets and PVB were determined by TGA of the composites (heating from room temperature to 700 °C in nitrogen followed by 30 minutes dwelling in synthetic air). Through equation (3), the results yield the ratio of volume fractions between alumina platelets and PVB in the composites. Substitution in (1) and determination of the mass density of the composite (geometrically, *i.e.* mass of a cuboid section divided by its outer dimensions) finally allows for calculation of volume fractions and, by using equation (2), porosity. All results are stated as average and range.

Quality of alignment was evaluated by use of scanning electron microscopy (SEM) on cross-sectional fracture surfaces of the sediments as well as by full-depth penetration synchrotron high-energy X-ray diffraction (HEXRD) along the diameter of the sediments at several heights. The latter is thereby meant to provide data averaged over the whole sample volume. It can thus confirm findings from SEM imaging with a far lower risk of biasing by detail selection and sample preparation.

SEM pictures of all samples were taken using secondary electrons of gold-sputtered samples at an acceleration voltage of 2 kV in high vacuum (≤10^−4^ Pa). Two samples, a non-pressed and a pressed sediment, both measuring 30 mm in diameter and containing 15.8 and 4.0 vol% PVB and 64.8 and 31.0% porosity, respectively, were measured using HEXRD. The measurements were made at experimental hutch 3 (EH3) at the Helmholtz-Zentrum Geesthacht beamline P07 (HEMS) of the PETRA III synchrotron storage ring at Deutsches Elektronen-Synchrotron (DESY) in Hamburg, Germany. An X-ray beam with size 0.1 mm (horizontally) × 0.5 mm (vertically) and an energy of 98.7 keV *(λ* = 0.012563 nm) impinged on a sample pellet which was placed vertically standing to the sample holder such that the beam travelled parallel to the pellet surface (fig. 3a). The detector was a two-dimensional Perkin Elmer 1621 detector with a pixel size of 0.2 mm x 0.2 mm at a sample-to-detector distance of 1400 mm. XRD patterns were recorded while moving the sample in steps of 0.1 mm in order to track the change in orientation degree within the polymer-stabilized top regions of the sediment samples. Measurement time for each XRD pattern was five seconds. X-ray transmission was measured using a photosensitive diode with exposure time of 0.5 s prior to the XRD measurements. As confirmed by transmission electron microscopy analysis (see fig. 6), the alumina particles used for sedimentation were α-Al_2_O_3_ single crystals (synthetic sapphire), in which the (0 0 1) plane is parallel to the plane of the flat particle surface. The orientation distributions of the particles were extracted from the radially averaged intensity of the (1 1 0) reflection (*d* = 0.23770 nm)^58^ of α-Al_2_O_3_. This represents the overall alignment well due to the statistical distribution of (1 1 0) facettes perpendicular to the (0 0 1) planes in every particle and the random rotation of all platelets around their [0 0 1] direction. Following other works with a similarly strong emphasis on alignment like those of Sequeira *et al.*^59^, Vainio *et al.*^60^, and Sklute *et al.*^61^, we selected the Hermans orientation parameter as one of our representations of the quality of alignment. As can be seen from this selection of references already, the Hermans orientation parameter is applicable to several material classes alike and even though there is some ambiguity in the definition on the Hermans orientation parameter depending on the method one uses and the definition one adopts, the values can be easily converted on the same scale. The Hermans orientation parameter was obtained from the intensity of the scattered beam *I*(*φ*)^62^,


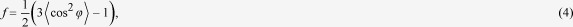


where the mean-square cosine is calculated from the intensity by integrating over the azimuth angle *φ*


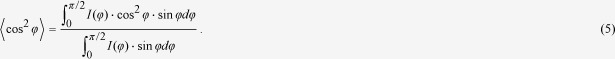


For the sake of convenience, we define *φ* = 0° to be at the centre of one of the 110 reflection maxima. For particle planes aligned perfectly along the pellet surface, *f* = 1, and for isotropic alignment, *f* = 0. When directly calculating *f* from experimental intensity, we noted that difficulties arise in determining the correct background level along the azimuth angle such that small changes in the selected background level caused large variations in *f*. To eliminate these uncertainties, which are mainly due to grain statistics, we calculated *f* from a fitted function. As the fitting function to the orientation distribution we used the generalized normal distribution^60^,^63^


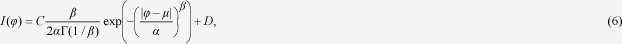


which gave a better fit than simple Gaussian. Here *α* is the scaling factor related to the width, *β* is the shape parameter determining the sharpness, and *μ* is the mean of the distribution. Γ denotes the gamma function and C is an overall scaling factor of the function and D represents the background level. The standard deviation is then calculated as^63^


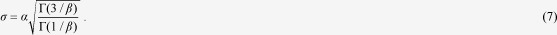


## Author Contributions

G.A.S., M.M. and A.S. motivated the research. S.B. developed the sedimentation process, prepared all samples and performed the characterization by SEM, rheology and means of sample composition. U.V. designed, conducted and interpreted the HEXRD measurements. All authors contributed extensively to the discussion of the results and reviewed the manuscript. S.B. wrote the main paper with U.V. contributing those parts concerning HEXRD measurements, their results and their discussion.

## Additional Information

**How to cite this article**: Behr, S. *et al*. Large-scale parallel alignment of platelet-shaped particles through gravitational sedimentation. *Sci. Rep.* **5**, 9984; doi: 10.1038/srep09984 (2015).
